# Children with heterozygous familial hypercholesterolaemia: routine lipoprotein(a) testing, earlier PCSK9 access, and pragmatic cascade screening

**DOI:** 10.1093/ehjopen/oeaf127

**Published:** 2025-10-05

**Authors:** Morgan Keogh

**Affiliations:** GKT School of Medical Education, King’s College London, Guy’s Campus, Great Maze Pond, London SE1 1UL, UK

**Keywords:** Familial hypercholesterolaemia, Children, Lipoprotein(a), PCSK9 inhibitors, Cascade screening, LDL-C

**This correspondence refers to ‘Management of children with heterozygous familial hypercholesterolaemia worldwide: a meta-analysis’, by I. Bytyçi *et al.*, https://doi.org/10.1093/ehjopen/oeaf001.**

I read with great interest the meta-analysis by Bytyçi *et al.* on the management of children with heterozygous familial hypercholesterolaemia (HeFH). The authors synthesize evidence on lipid-lowering therapy efficacy, safety, and goal attainment, providing timely confirmation that intensive LDL-cholesterol (LDL-C) reduction is both achievable and well tolerated in paediatric practice. Their work reinforces the importance of treatment escalation when LDL-C targets are not achieved.^1^

Building on these findings, I propose three practical refinements that could further strengthen routine care pathways for children with HeFH: (i) incorporation of lipoprotein(a) [Lp(a)] testing into baseline assessment, (ii) earlier consideration of PCSK9 inhibitors in selected children, and (iii) systematic cascade screening centred on the family unit.

1. Lp(a) testing as a standard baseline measure.
  Elevated Lp(a) is a powerful independent risk factor for atherosclerotic cardiovascular disease. The European Atherosclerosis Society consensus recommends at least one lifetime measurement in adults, and targeted testing in youth with inherited dyslipidaemia or a family history of premature atherosclerotic cardiovascular disease (ASCVD), situations that frequently apply in HeFH clinics. Including a one-time Lp(a) test at the initial diagnostic panel would refine individualized risk discussions and support decisions on treatment intensity.^2^ It would also reduce the need for repeated sampling unless assay availability changes or new clinical questions arise.
2. Earlier access to PCSK9 inhibitors.
  Randomized controlled data in paediatric HeFH demonstrate that evolocumab produces substantial additional LDL-C reduction beyond statins and ezetimibe, with a safety profile comparable to placebo.^3^ While access varies across health systems, consideration of PCSK9 therapy at an earlier stage for children with very high baseline LDL-C, intolerance to high-intensity statins, or persistent elevation during puberty could help reduce cumulative LDL exposure at a critical time in vascular development. Such an approach would align clinical care with the treat-to-target principle already highlighted by Bytyçi *et al.*
3. Strengthening cascade screening.
  Heterozygous familial hypercholesterolaemia is a familial condition, yet systematic cascade testing is inconsistently implemented. Embedding clear workflows, such as family invitation letters, electronic health record prompts, or clinic-initiated contact with first-degree relatives, would improve yield and ensure earlier detection. This pragmatic strategy balances international debates about universal childhood lipid screening by focusing resources where risk is highest. Importantly, it also improves family engagement and shared responsibility for health.

Operationalizing in practice.

A practical model for paediatric lipid clinics would be the following: (i) add a one-time Lp(a) to the initial lipid panel; (ii) adopt a stepped ‘treat-to-target’ ladder: statin → statin plus ezetimibe → PCSK9 inhibitor if goals remain unmet; and (iii) implement structured cascade workflows with clear roles and responsibilities. Embedding concise counselling on adherence, lifestyle, and reproductive risk during adolescence would further support safe and sustained lipid management.

In summary, the meta-analysis by Bytyçi *et al.* consolidates the evidence for effective and safe LDL-C reduction in paediatric HeFH. Building on this strong foundation, introducing routine Lp(a) testing, enabling earlier access to PCSK9 inhibitors for selected children, and formalizing cascade screening can help accelerate target attainment, reduce lifetime LDL burden, and empower families in the care process.
